# Supplementary material for: Secure human action recognition by encrypted neural network inference
Source: Nat Commun. 2022 Aug 15;13:4799. doi: 10.1038/s41467-022-32168-5 (PMC9378731; doi:10.1038/s41467-022-32168-5)
Supplement: Supplementary file 1 — Supplementary Information [file 41467_2022_32168_MOESM1_ESM.pdf]

Supplementary Notes for "Secure Human Action Recognition by Encrypted Neural Network Inference"

Miran Kim[1,2,*], Xiaoqian Jiang[3], Kristin Lauter[4], Elkhan Ismayilzada[5], and Shayan Shams[6,*]

[1]Department of Mathematics, Hanyang University, Seoul, Republic of Korea.
[2]Department of Computer Science, Hanyang University, Seoul, Republic of Korea.
[3]Center for Secure Artificial intelligence For hEalthcare (SAFE), School of Biomedical Informatics, University of Texas Health Science Center, Houston, TX, USA.
[4]Meta AI Research, Seattle, WA, USA.
[5]Department of Computer Science and Engineering, Ulsan National Institute of Science and Technology, Ulsan, Republic of Korea.
[6]Department of Applied Data Science, San Jose State University, San Jose, CA, USA.
[*]Corresponding author(s): miran@hanyang.ac.kr, Shayan.Shams@sjsu.edu

Supplementary Note 1: The CKKS Cryptosystem

Fully homomorphic encryption (FHE) is a cryptosystem that allows for operations on encrypted inputs without decryption. Throughout the paper, we assume that N is a power-of-two integer and $R = \mathbb{Z}[X]/(X^N + 1)$. We write $R_q = R/(q \cdot R)$ for the residue ring of R modulo an integer q. The CKKS scheme[1] is a leveled HE scheme with support for approximate fixed-point arithmetic. Given a computational level budget L, we assume $q = \prod_{i=0}^{L} p_i$ for some integers p_i and let $q_\ell = \prod_{i=0}^{\ell} p_i$. The following is a simple description of CKKS based on the ring learning with errors (RLWE) problem.

• $\underline{\mathsf{Setup}(1^\lambda)}$: For a given security parameter λ, set the RLWE dimension N, ciphertext modulus q, key distribution χ and error distribution ψ over R. Return the public parameter $pp = (n, q, \chi, \psi)$.

• $\underline{\mathsf{KeyGen}(pp)}$: Sample the secret key $s \leftarrow \chi$, a random ring element $a \leftarrow U(R_q)$, and an error $e \leftarrow \psi$. Set $b = -s \cdot a + e \pmod q$. Set the public key $\mathsf{pk} = (b, a)$ and the secret key $\mathsf{sk} = (1, s)$.

• $\underline{\mathsf{Enc}(m; \mathsf{pk})}$: Let $m \in R$ be an input plaintext. Sample $v \in \chi$ and $e_0, e_1 \leftarrow \psi$. Return the ciphertext $\mathsf{ct} = (c_0, c_1) \in R_q^2$ where $c_0 = v \cdot b + m + e_0 \pmod q$ and $c_1 = v \cdot a + e_1 \pmod q$.

• $\underline{\mathsf{Dec}(\mathsf{ct}; \mathsf{sk})}$: Let $\mathsf{ct} = (c_0, c_1) \in R_{q_\ell}^2$ be a ciphertext. Return $\langle \mathsf{ct}, \mathsf{sk} \rangle \pmod{q_\ell}$.

• $\underline{\mathsf{Add}(\mathsf{ct}_1, \mathsf{ct}_2)}$: Given two ciphertexts $\mathsf{ct}_i \in R_{q_\ell}^2$, return the ciphertext $\mathsf{ct}' = \mathsf{ct}_1 + \mathsf{ct}_2 \pmod{q_\ell}$.

• $\underline{\mathsf{Mult}(\mathsf{ct}_1, \mathsf{ct}_2)}$: Given two ciphertexts $\mathsf{ct}_i \in R_{q_\ell}^2$, compute $\mathsf{ct} = \mathsf{ct}_1 \otimes \mathsf{ct}_2 \pmod{q_\ell}$ and return the ciphertext $\mathsf{ct}' \in R_{q_\ell}^2$ such that $\langle \mathsf{ct}', \mathsf{sk} \rangle \approx \langle \mathsf{ct}, (1, s, s^2) \rangle \pmod{q_\ell}$.

• $\underline{\mathsf{Rescale}(\mathsf{ct})}$: Given a ciphertext $\mathsf{ct} = (c_0, c_1) \in R_{q_\ell}^2$, compute $c_i' = \lfloor p_\ell^{-1} \cdot c_i \rceil$ for $i = 0, 1$, and return the ciphertext $\mathsf{ct}' = (c_0', c_1') \in R_{q_{\ell-1}}^2$.

For $0 \le \ell \le L$, a ciphertext (or a plaintext) is at level ℓ if its coefficients are in q_ℓ. The CKKS scheme supports the *ciphertext packing technique* to encrypt vectors of elements and perform parallel homomorphic operations in a SIMD manner (e.g., element-wise addition and multiplication over vectors). A ciphertext can hold up to $N/2$ plaintext values. Furthermore, it allows one to perform the rotation operation, denoted by $\rho^\ell(\mathsf{ct})$, which transforms an encryption ct of $\mathbf{v} = (v_0, \ldots, v_{N/2-1})$ into an encryption of $\rho^\ell(\mathbf{v}) := (v_\ell, \ldots, v_{N/2-1}, v_0, \ldots, v_{\ell-1})$. We note that ℓ can be either positive or negative, and a rotation by $(-\ell)$ is the same as a rotation by $(N/2 - \ell)$. We refer to the reader to the CKKS scheme[1,2] for further details.

Supplementary Note 2: Homomorphic Convolutions

In an 1D-CNN, we define the rotation amounts $r_i^{\text{(pre)}}$ ad $r_\ell^{\text{(post)}}$ as

$$r_i^{\text{(pre)}} = i - 1, \tag{1}$$
$$r_\ell^{\text{(post)}} = 2^{\ell-1}, \tag{2}$$

for $2 \le i \le n_P$, $1 \le \ell \le \lceil \log n_P \rceil$. Here, $r_i^{\text{(pre)}}$ are $r_\ell^{\text{(post)}}$ are used for the preprocessing and postprocessing steps, respectively. In a 2D-CNN, we define the rotation amounts as follows:

$$r^{\text{(pre)}}_{m+k\sqrt{n_P}} = (m-1) + k \cdot w, \tag{3}$$
$$r^{\text{(post)}}_{m'+k'\log\sqrt{n_P}} = \begin{cases} 2^{m'-1}, & \text{if } k' = 0, \\ 2^{m'-1} \cdot w, & \text{if } k' = 1; \end{cases} \tag{4}$$

for $1 \le m \le \sqrt{n_P}$, $0 \le k < \sqrt{n_P}$, $1 \le m' \le \lceil \log \sqrt{n_P} \rceil$, and $k' = 0, 1$. Algorithm 1 describes the fast homomorphic convolution.

Algorithm 1 Fast homomorphic convolution

function: $\textsc{FastHomConv}(\{\mathsf{ct}_j\}, \{\mathsf{pt.F}_{i,j,k,\ell}\}, \mathsf{pt}_{\mathsf{zone}}, n_P)$
Input: $\{\mathsf{ct}_u\}$: punctured ciphertexts $(1 \le u \le n_{\text{in}})$; $\{\mathsf{pt.F}_{i,j,k,\ell}\}$: plaintexts for the weights of the filters $(1 \le i \le n_{\text{in}}/n_P, 1 \le j \le n_{\text{out}}, |k| \le f, 0 \le \ell \le \bar{c}_{\text{in}})$; $\mathsf{pt}_{\mathsf{zone}}$: a plaintext that encodes one in the valid entries of the corresponding input ciphertexts and zero otherwise; n_P: a load number

[Pre-processing step]
for $1 \le v \le n_{\text{in}}/n_P$ **do**
  for $1 \le u \le n_P$ **do**
    $\mathsf{ct.pack}_u \leftarrow \mathsf{MultPlain}(\mathsf{ct}_{u+(v-1)\cdot n_P}, \mathsf{pt}_{\mathsf{zone}})$
  end for
  for $2 \le u \le n_P$ **do**
    $\mathsf{ct.pack}_1 \leftarrow \mathsf{Add}(\mathsf{ct.pack}_1, \rho^{-r_u^{\text{(pre)}}}(\mathsf{ct.pack}_u))$
  end for
  $\mathsf{ct.pre}_v \leftarrow \mathsf{Rescale}(\mathsf{ct.pack}_1)$
end for
[Ordinary homomorphic convolution]
for $1 \le j \le n_{\text{out}}$ **do**
  $\mathsf{ct.conv}_j \leftarrow \mathsf{HE\text{-}Conv}_j(\mathsf{ct.pre}_1, \ldots, \mathsf{ct.pre}_{n_{\text{in}}/n_P}, \{\mathsf{pt.F}_{i,j,k,\ell}\})$
end for
[Post-processing step]:
for $1 \le j \le n_{\text{out}}$ **do**
  $\mathsf{ct.res}_j \leftarrow \mathsf{ct.conv}_j$
  for $1 \le \ell \le \lceil \log(n_P) \rceil$ **do**
    $\mathsf{ct.res}_j \leftarrow \mathsf{Add}(\mathsf{ct.res}_j, \rho^{r_\ell^{\text{(post)}}}(\mathsf{ct.res}_j))$
  end for
end for
return $\{\mathsf{ct.res}_j\}_{1 \le j \le n_{\text{out}}}$

Supplementary Note 3: Non-convolutional Layers

Collapsing consecutive layers. For each feature map, suppose that we are given the following parameters : (i) learned bias b of the convolution layer; (ii) Batch normalization (BN) statistics (μ, σ, γ, β); (iii) coefficient parameters of an activation function (a_0, a_1, a_2) such that the activation is defined as $x \mapsto a_0 + a_1 x + a_2 x^2$; (iv) window size (s_1, s_2) of the average pooling layer. We recall the BN of Ioffe and Szegedy[3]. We denote by x an output value of a convolutional layer of the network without adding the bias term. Then it is updated as follows:

$$z = \gamma\left(\frac{(x+b)-\mu}{\sigma}\right) + \beta = d_0 + d_1 \cdot x, \tag{5}$$

where $d_1 = \gamma/\sigma$ and $d_0 = \beta + d_1(b-\mu)$. Afterward, the output is activated as

$$a_0 + a_1 z + a_2 z^2 = (a_0 + a_1 \cdot d_0 + a_2 \cdot d_0^2) + (a_1 \cdot d_1 + 2 \cdot a_2 \cdot d_0 \cdot d_1)x + (a_2 \cdot d_1^2)x^2. \tag{6}$$

In the end, we sum up all the parameters. As a result, the composite of the three operations is expressed as $x \mapsto c_0 + c_1 \cdot x + c_2 \cdot x^2$ where

$$\begin{aligned} c_0 &= \frac{a_0 + a_1 \cdot d_0 + a_2 \cdot d_0^2}{s_1 \cdot s_2}, \\ c_1 &= \frac{a_1 \cdot d_1 + 2 \cdot a_2 \cdot d_0 \cdot d_1}{s_1 \cdot s_2}, \\ c_2 &= \frac{a_2 \cdot d_1^2}{s_1 \cdot s_2}. \end{aligned} \tag{7}$$

To be precise, we let $c^t_{n,m}$ be the m-th coefficient of the n-th feature map at the t-th block of the network. For $1 \le j \le n^t_O$, we denote by $\mathsf{pt.coeff}^t_{j,m}$ a plaintext polynomial of the vector obtained by concatenating the m-th coefficients $c^t_{n,m}$'s for $\bar{c}^t_{\text{out}} \cdot (j-1) < n \le \bar{c}^t_{\text{out}} \cdot j$.

Fully-connected layer. Let $\mathbf{W}$ be a weight matrix of size $d_{\text{out}} \times d_{\text{in}}$. Suppose that a length-$d_I$ output vector $\mathbf{v}$ of the global average pooling layer is given as multiple ciphertexts that have c values of the vector in a sparse way. We split $\mathbf{v}$ into sub-strings $\mathbf{v}_i$'s with the same length c. Accordingly, we split the original matrix $\mathbf{W}$ into $(d_{\text{out}} \times c)$-sized smaller blocks $\mathbf{W}_i$ and perform a matrix-vector multiplication on the sub-matrices: $\mathbf{W} \cdot \mathbf{v} = \sum_{1 \le i \le d_{\text{in}}/c} \mathbf{W}_i \cdot \mathbf{v}_i$. We apply the diagonal encoding method of matrix-vector multiplication[4] to the computation on the sub-matrices: it puts a square matrix in a diagonal order, multiplies each matrix with the rotated input vector, and then sums all output vectors to obtain the result. Consequently, the output ciphertext has d_{out} predicted results. We use $\mathsf{padZeros}(\mathbf{A}; \mathbf{v}, \mathsf{dir})$ to pad a matrix $\mathbf{A}$ with zeros in the direction specified by dir, where $\mathbf{v}$ is a vector of non-negative integers that specify the amount of padding to add and the dimension along which to add it. The direction can be specified as one of the following values: L (left), R (right), U (upper), and B (bottom). To be specific, the result of matrix-vector multiplication is obtained by taking the first d_{out} entries of the vector

$$\sum_{\substack{1 \le i \le d_I/c, \\ 1 \le \ell \le c}} \mathsf{Diag}_\ell(\mathsf{padZeros}(\mathbf{W}_i; c - d_{\text{out}}, \mathsf{B})) \odot \rho^\ell(\mathbf{v}_i), \tag{8}$$

where $\mathsf{Diag}_\ell(\mathbf{A})$ indicates the ℓ-th diagonal component of a square matrix $\mathbf{A}$ and $\odot$ denotes an element-wise multiplication over vectors. By the assumption, $\mathbf{v}_i$ is given as a ciphertext ct_i that represents the entries in a sparsely-packed way. For the sake of brevity, let $\mathbf{W}_{i\ell} = \mathsf{Diag}_\ell(\mathsf{padZeros}(\mathbf{W}_i; c - d_{\text{out}}, \mathsf{B}))$. Therefore, Equation (8) can be securely computed as follows:

$$\sum_{i,\ell} \mathsf{MultPlain}(\rho^{\mathsf{dist}_v \cdot \ell}(\mathsf{ct}_i), \mathbf{W}_{i\ell}), \tag{9}$$

where dist_v indicates an actual distance of two valid entries of $\mathbf{v}_i$ over plaintext slots. Equation (9) requires $(d_{\text{in}}/c) \cdot c = d_{\text{in}}$ rotations. Furthermore, the number of rotations can be reduced to c by reformulating the equation as follows:

$$\sum_\ell \rho^{\mathsf{dist}_v \cdot \ell}\Big(\sum_i \mathsf{MultPlain}(\mathsf{ct}_i, \rho^{-\mathsf{dist}_v \cdot \ell}(\mathbf{W}_{i\ell}))\Big). \tag{10}$$

Supplementary Note 4: Implementation Optimizations

Adjusted level-aware encoding method. We investigate how ciphertext levels change during homomorphic evaluation of K-layered convolutional network models, and deduce the optimized encoding levels of weight parameters. We start with the network input ciphertext ct_X at level L in the HEAR system. Each convolutional layer of the network has a depth of one plaintext-ciphertext multiplication, and the collapsed layer of BN and activation requires a depth of one plaintext-ciphertext multiplication and ciphertext-ciphertext multiplication. Finally, the FC layer has a depth of one plaintext-ciphertext multiplication. Let $\mathsf{pt.F}_t$ be plaintext polynomials of the kernels at the t-th convolutional layer. Let $\mathsf{pt.coeff}_t^k$ be plaintext polynomials of the coefficient of degree-k approximation of the t-th activation. We use $\mathsf{pt.W}$ to denote plaintext polynomials of the weight matrix at the FC layer. Then we get the following minimum encoding levels for the model parameters:

- $\mathsf{level}(\mathsf{pt.F}_t) = L - 3 \cdot (t-1)$,
- $\mathsf{level}(\mathsf{pt.coeff}_t^k) = \begin{cases} \mathsf{level}(\mathsf{pt.F}_t) - 3, & \text{if } k = 0; \\ \mathsf{level}(\mathsf{pt.F}_t) - 2, & \text{otherwise,} \end{cases}$
- $\mathsf{level}(\mathsf{pt.W}) = \mathsf{level}(\mathsf{pt.F}_K) - 3$.

It suffices to satisfy the inequality of $\mathsf{level}(\mathsf{pt.W}) = (L - 3 \cdot (K-1)) - 3 \geq 1$. Therefore, we deduce that the level of the input ciphertext L is set to $3 \cdot K + 1$. Our action recognition network has a stack of three layers, so we have $L = 10$. In the context of the Fast-HEAR system, we need constant zero-one vectors to remove junk entries right after pooling layers, so the encoding levels for the kernels are $\mathsf{level}(\mathsf{pt.F}_t) = L - 4 \cdot (t-1)$. As a result, the level of the input ciphertext L is set to $4 \cdot K$, which is 12 in the action recognition network. Supplementary Figure 1 shows how ciphertext levels change during homomorphic computation of the HEAR and Fast-HEAR systems.

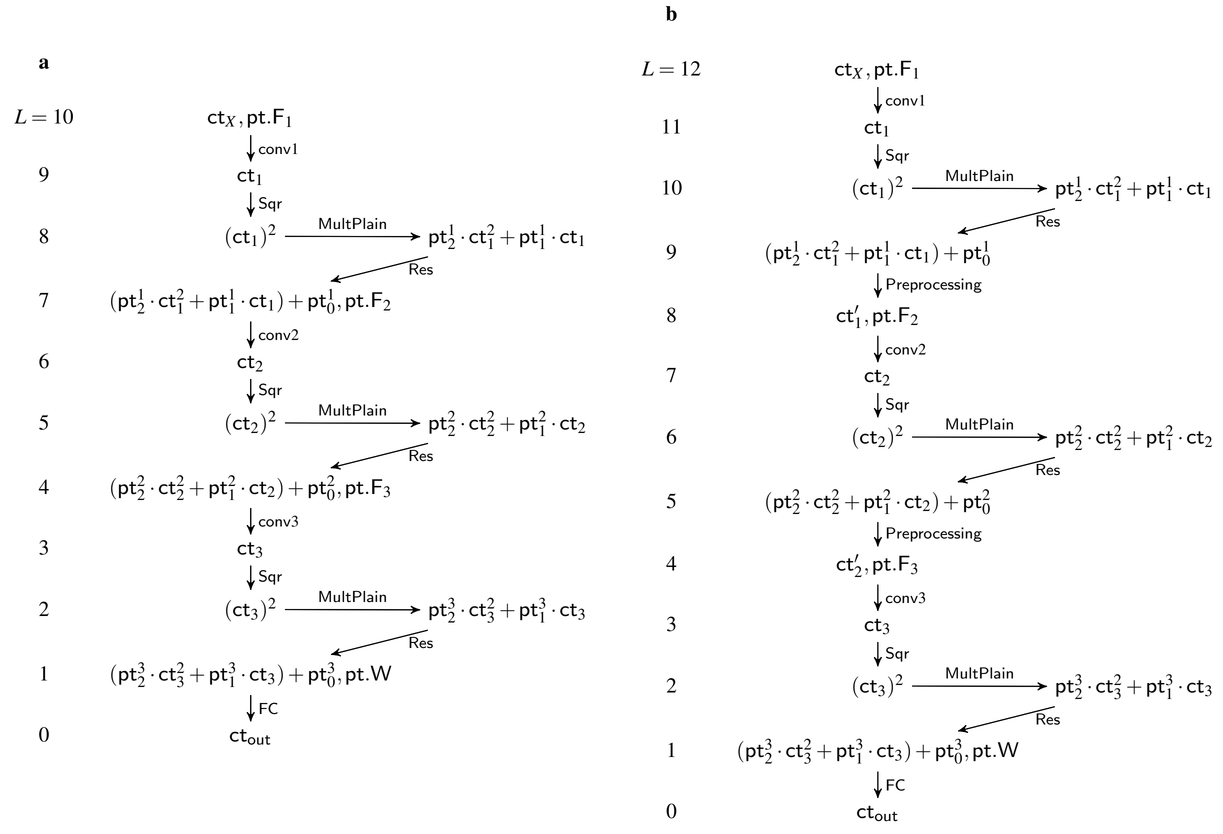

Supplementary Figure 1. An illustration of changes of ciphertext levels during homomorphic computation. The numbers on the left sides indicate the ciphertext levels at each computation phase. For the sake of brevity, we let $\mathsf{pt}_t^k = \mathsf{pt.coeff}_t^k$. We denote by $\mathsf{conv}i$ the i-th homomorphic convolution, Sqr a squaring operation, $\mathsf{MultPlain}$ a plaintext-ciphertext multiplication, and Res a rescaling operation. **a** Evaluation procedure of HEAR on our action recognition network. **b** Evaluation procedure of Fast-HEAR on our action recognition network.

Supplementary Table 1. The computational cost of the ordinary and fast convolutional layers over the giant-step strategy. Rot denotes the ordinary rotation which cannot benefit from hoisting, and Hoisted Rot denotes multiple rotations on the same input ciphertext that can benefit from the hoisting optimization. H-Rot$_k$ requires $(k-1)$ rotations on an input ciphertext. The third and sixth rows are obtained by counting the homomorphic operations to conduct the convolutional layers. The specified numbers of the conv2 and conv3 rows are calculated when evaluating the 2D-CNN-128 network. In our experiment, we set $\bar{c}_{\text{in}} = 16$ at the second and third convolutional layers.

Method	Layer	Homomorphic operations count			
		Rot	Hoisted Rot	MultPlain	Rescale
Ordinary homomorphic convolution	conv	$8 \cdot n_{\text{out}}$	$n_{\text{in}} \cdot \text{H-Rot}_{\bar{c}_{\text{in}}}$	$f \cdot \bar{c}_{\text{in}} \cdot n_{\text{in}} \cdot n_{\text{out}}$	n_{out}
	conv2	$8 \cdot 16 = 128$	$8 \cdot \text{H-Rot}_{16}$	$9 \cdot 16 \cdot 8 \cdot 16 = 18432$	16
	conv3	$8 \cdot 32 = 256$	$16 \cdot \text{H-Rot}_{16}$	$9 \cdot 16 \cdot 16 \cdot 32 = 73728$	32
Fast homomorphic convolution	conv	$(n_P - 1) \cdot (n_{\text{in}}/n_P) + n_{\text{out}} \cdot (\log n_P + 8)$	$(n_{\text{in}}/n_P) \cdot \text{H-Rot}_{\bar{c}_{\text{in}}}$	$n_{\text{in}} + f \cdot \bar{c}_{\text{in}} \cdot (n_{\text{in}}/n_P) \cdot n_{\text{out}}$	$n_{\text{out}} + (n_{\text{in}}/n_P)$
	conv2	$(4-1) \cdot (8/4) + 16 \cdot (\log 4 + 8) = 166$	$2 \cdot \text{H-Rot}_{16}$	$8 + 9 \cdot 16 \cdot (8/4) \cdot 16 = 4616$	18
	conv3	$(16-1) \cdot 1 + 32 \cdot (\log 16 + 8) = 399$	H-Rot_{16}	$16 + 9 \cdot 16 \cdot (16/16) \cdot 32 = 4624$	33

Supplementary Table 2. Homomorphic encryption parameters for HEAR and Fast-HEAR. L indicates the required number of levels to implement the network architecture. q is the ciphertext modulus of a freshly encrypted encrypted input, and q_0 is the output ciphertext modulus. Δ_{msg} is the scaling factor for an input tensor and weight parameters. Δ_{mask} is the scaling factor for multiplicative masking vectors in Fast-HEAR. The special modulus P is chosen to reduce the noise growth during homomorphic operations.

Method	HE Parameters					
	L	$\log q$	$\log q_0$	$\log \Delta_{\text{msg}}$	$\log \Delta_{\text{mask}}$	$\log P$
HEAR	10	343	33	31	-	33
Fast-HEAR	12	399	33	31	28	33

Theoretical complexity of homomorphic convolution. In Supplementary Table 1, we provide a theoretical analysis of computational costs of the ordinary and fast convolution layers that use the giant-step strategy. As an example, we provide concrete numbers of homomorphic operations for secure inference on the 2D-CNN-128 network.

Encryption parameter selection. We multiply a scale factor of Δ to plaintexts and perform the rescaling procedure by a factor of Δ on ciphertexts after each multiplication to maintain the precision of the plaintext. Then, a ciphertext modulus is reduced by $\log \Delta$ bits after multiplication, or a multiplication operation consumes one level. It requires 10 levels to implement the network architecture of HEAR, whereas Fast-HEAR needs two additional levels of plaintext-ciphertext multiplications for the preprocessing step. In the HEAR system, the ciphertext modulus $q = \prod_{i=0}^{10} p_i$ is defined as $\log p_i \approx \log \Delta_{\text{msg}} = 31$ for $1 \le i \le 10$. The Fast-HEAR system performs a ciphertext-plaintext multiplication with the masking vector pt_{zone} (in Algorithm 1) at levels 5 and 9, so the ciphertext modulus $q = \prod_{i=0}^{12} p_i$ is as follows: $\log p_5 \approx \log p_9 \approx \log \Delta_{\text{mask}} = 28$ and $\log p_i \approx \log \Delta_{\text{msg}} = 31$ otherwise.

We use uniform ternary secret distribution (i.e., the uniform distribution over the set of polynomials whose coefficients are in $\{-1, 0, 1\}$). Each coefficient of an error is drawn according to the discrete Gaussian distribution centered at zero with standard deviation $\sigma = 3.2$. In the end, we take the ciphertext ring dimension $N = 2^{14}$ to ensure 128 bits of security against the known attacks on the LWE problem from the LWE estimator[5] and HE security standard white paper[6]. The encryption parameters are summarized in Supplementary Table 2.

Supplementary Note 5: Experimental Results of Secure Inference

Supplementary Figure 2 shows the execution time on Fast-HEAR with various evaluation strategies of homomorphic convolution. Supplementary Figure 3 shows the timing results of secure inference of HEAR and Fast-HEAR with respect to various number of available threads. Our implementation exploited multiple cores, when available, and these results show that at least up to 16 cores, the speedups scale linearly with the number of cores in all methods.

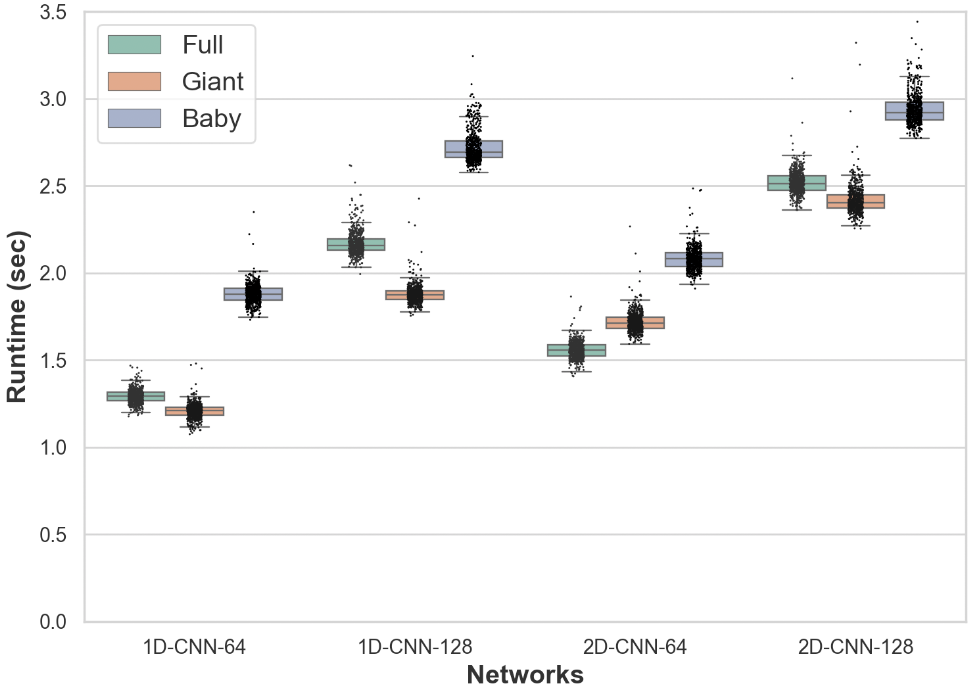

Supplementary Figure 2. Average running time for secure inference on $n = 608$ independent samples from the test set over various neural network models with various evaluation strategies in Fast-HEAR. The boxplot displays the median values with the first and third quartile, and the whiskers boundaries represent the largest and smallest data values no more than 1.5 times the interquartile range (IQR) from the corresponding hinge.

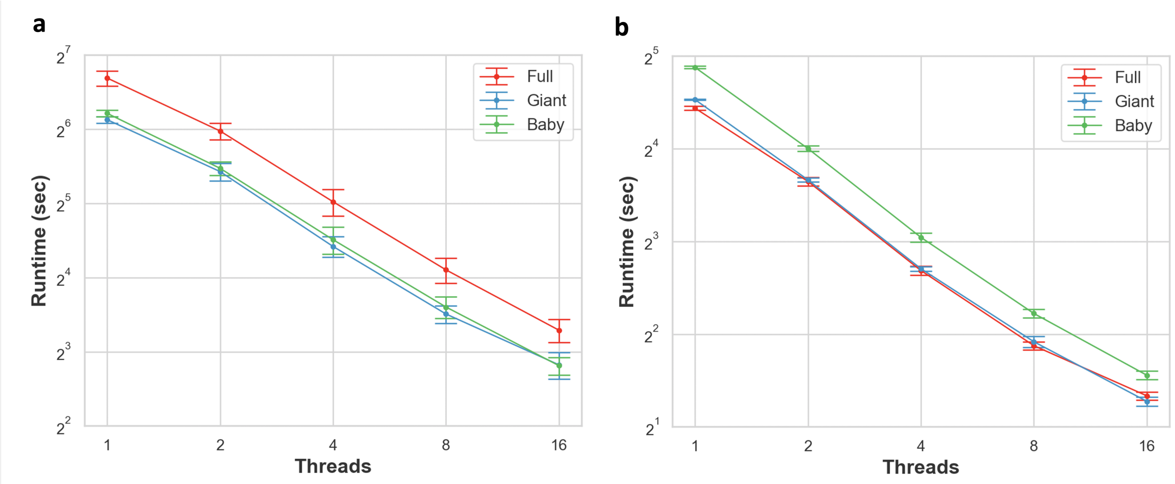

Supplementary Figure 3. Average running time for secure inference on the 2D-CNN-128 network with respect to various threads (log-log scale). Data are presented as mean±standard deviation from n=608 independent samples on the test set. **a** Experimental result of HEAR. **b** Experimental result of Fast-HEAR.

References

1. Cheon, J. H., Kim, A., Kim, M. & Song, Y. Homomorphic encryption for arithmetic of approximate numbers. In *Advances in Cryptology–ASIACRYPT 2017: 23rd International Conference on the Theory and Application of Cryptology and Information Security*, 409–437 (Springer, 2017).
2. Cheon, J. H., Han, K., Kim, A., Kim, M. & Song, Y. A full RNS variant of approximate homomorphic encryption. In *International Conference on Selected Areas in Cryptography*, 347–368 (Springer, 2018).
3. Ioffe, S. & Szegedy, C. Batch normalization: Accelerating deep network training by reducing internal covariate shift. In *International Conference on Machine Learning*, 448–456 (PMLR,2015).
4. Halevi, S. & Shoup, V. Algorithms in HElib. In *Advances in Cryptology–CRYPTO 2014*, 554–571 (Springer, 2014).
5. Albrecht, M. R., Player, R. & Scott, S. On the concrete hardness of learning with errors. *J. Math. Cryptol.* **9**, 169–203 (2015).
6. Albrecht, M. *et al.* Homomorphic encryption security standard. Tech. Rep., HomomorphicEncryption.org, Toronto, Canada (2018).
