# Supplementary figures and images for: Secure human action recognition by encrypted neural network inference
Source: Nat Commun. 2022 Aug 15;13:4799. doi: 10.1038/s41467-022-32168-5 (PMC9378731; doi:10.1038/s41467-022-32168-5)

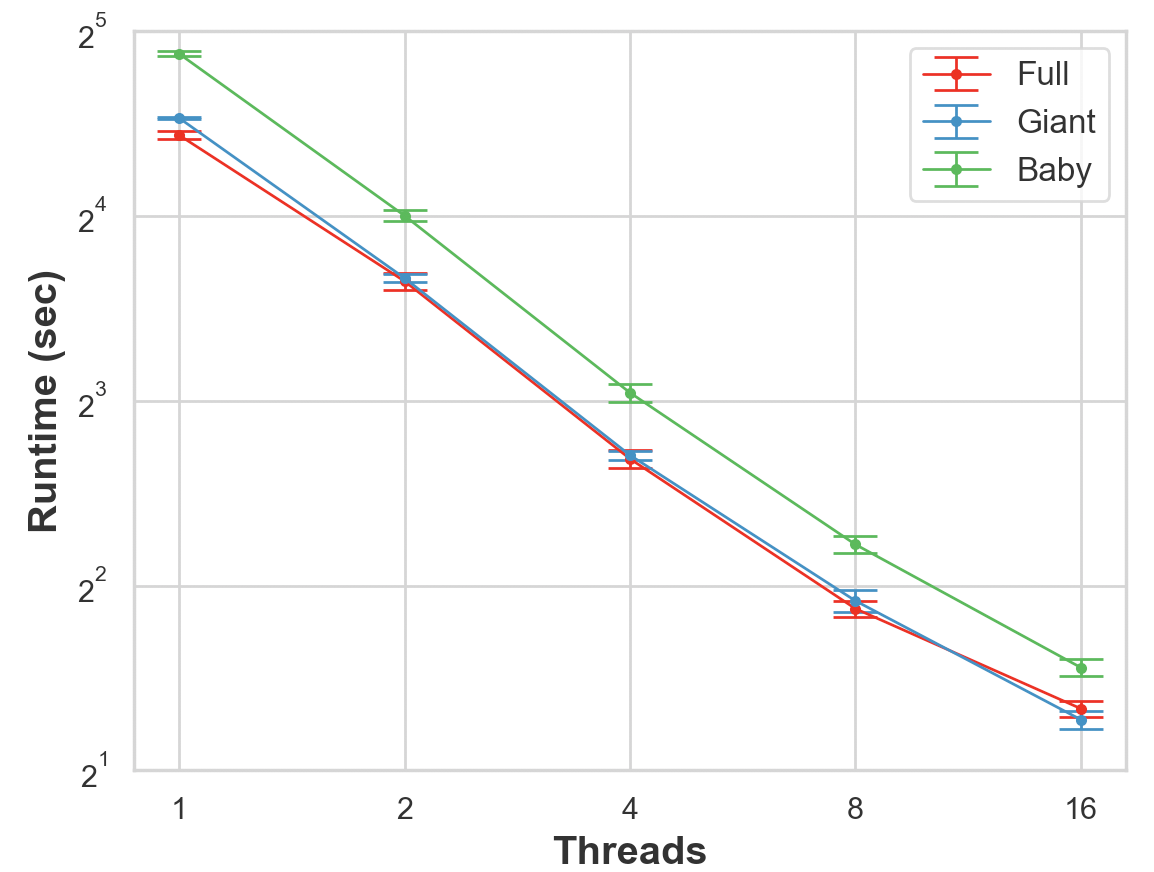

Supplement: Supplementary file 4 — Source data [file 41467_2022_32168_MOESM4_ESM.zip › SOURCE_DATA/SupplementaryFigure3/sfig3b.png]

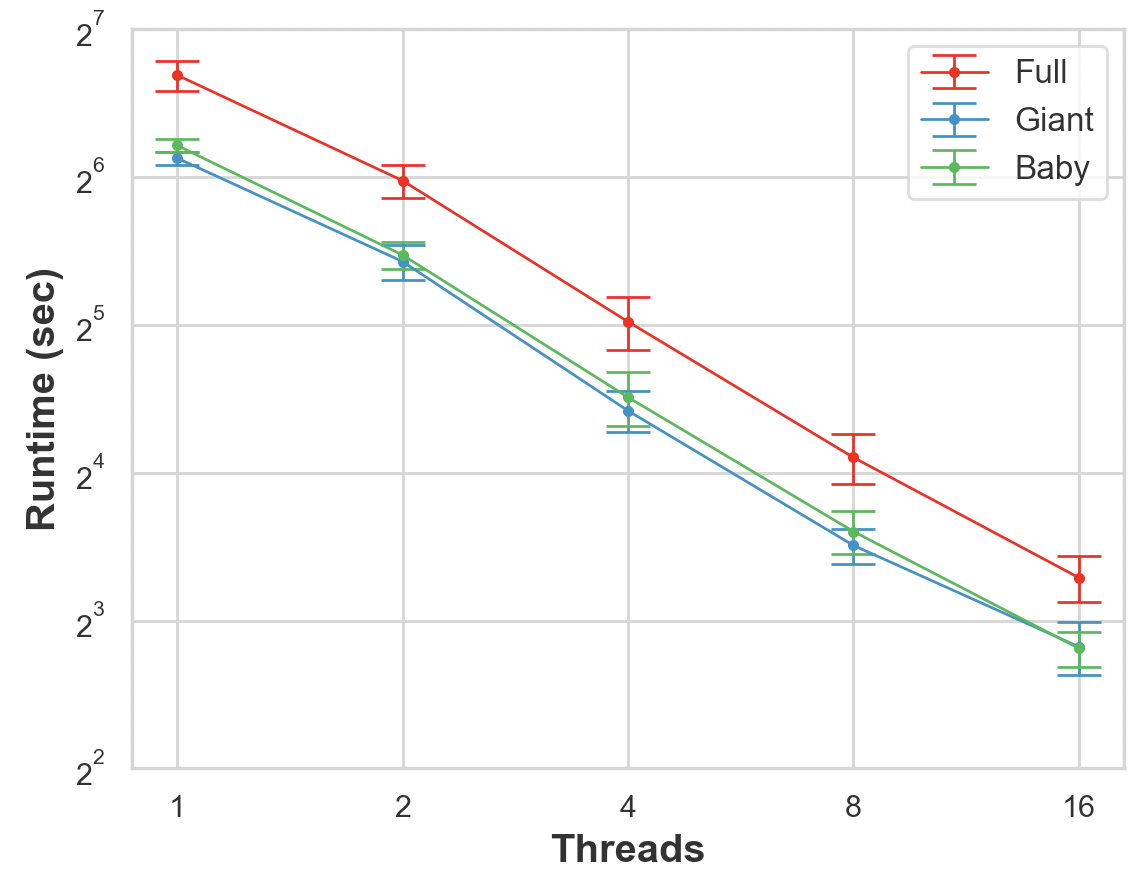

Supplement: Supplementary file 4 — Source data [file 41467_2022_32168_MOESM4_ESM.zip › SOURCE_DATA/SupplementaryFigure3/sfig3a.png]

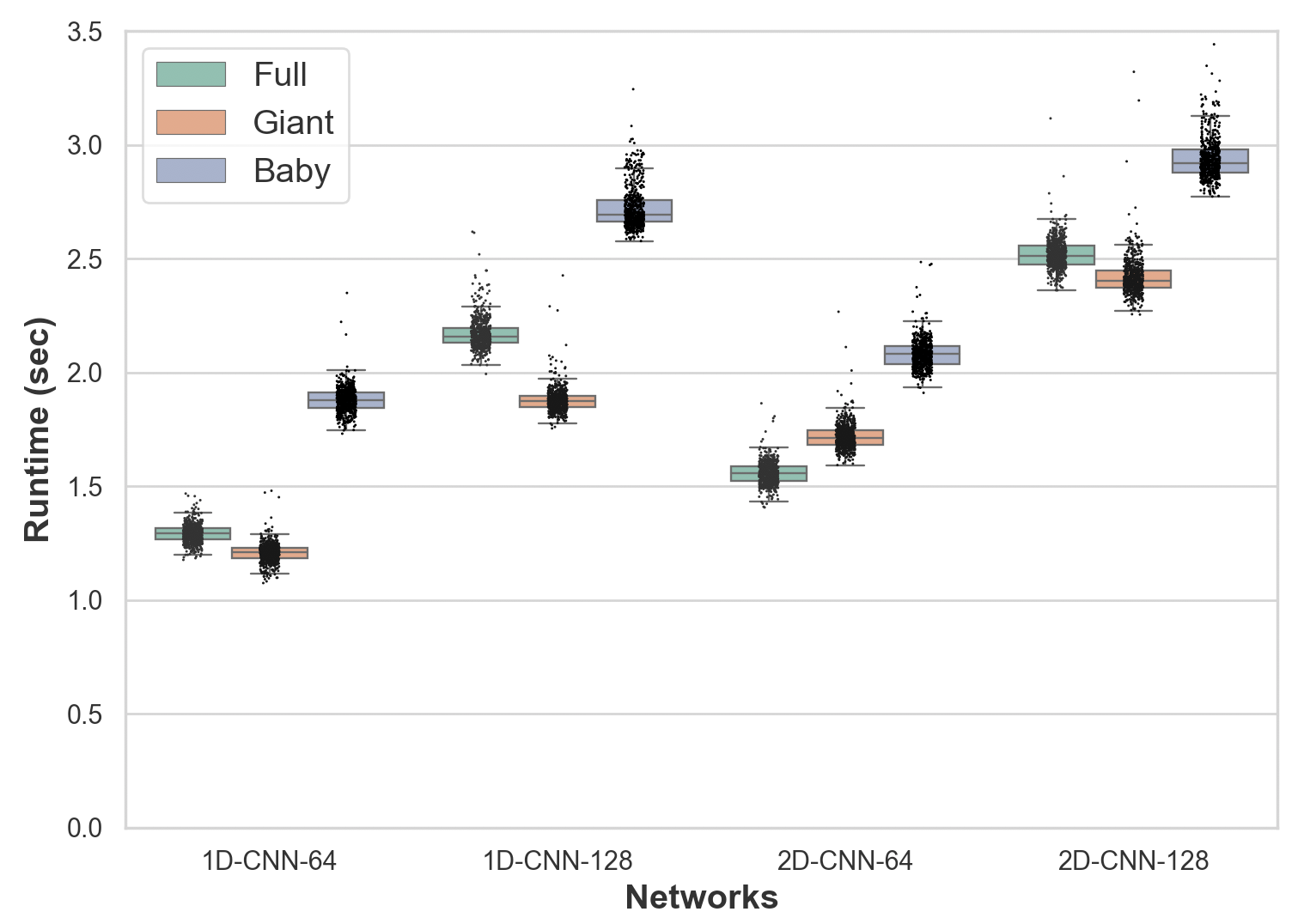

Supplement: Supplementary file 4 — Source data [file 41467_2022_32168_MOESM4_ESM.zip › SOURCE_DATA/SupplementaryFigure2/sfig2.png]

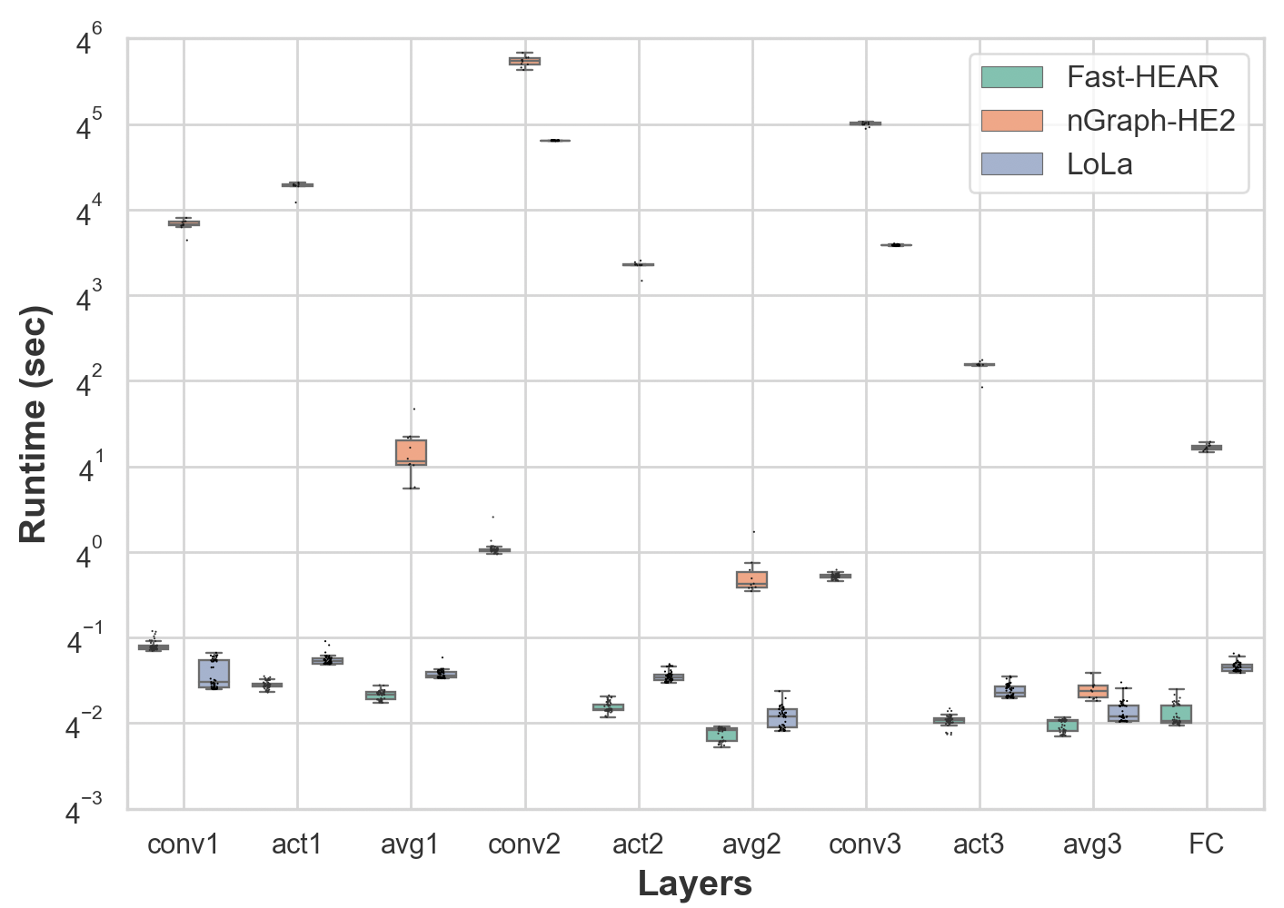

Supplement: Supplementary file 4 — Source data [file 41467_2022_32168_MOESM4_ESM.zip › SOURCE_DATA/Figure5_SOTA/5b_evaluation_detail/fig5b.png]

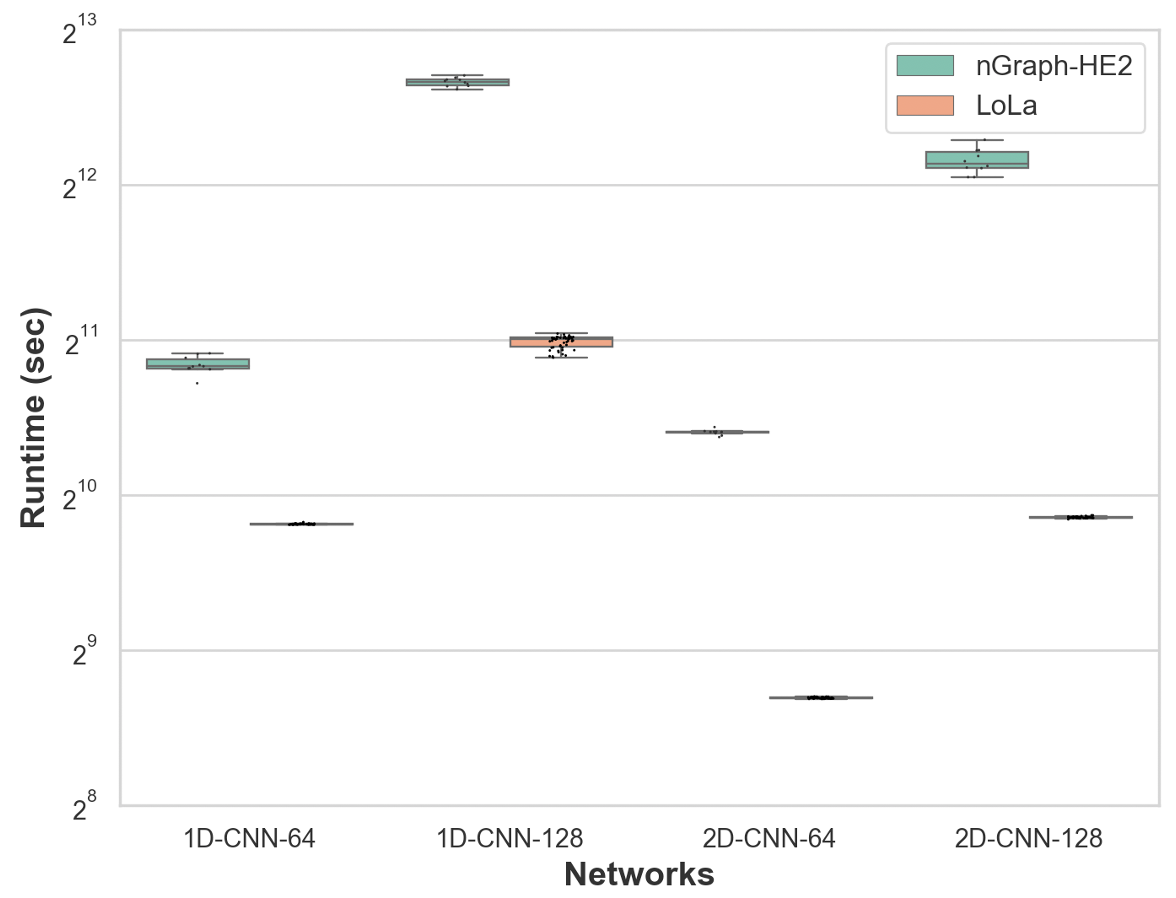

Supplement: Supplementary file 4 — Source data [file 41467_2022_32168_MOESM4_ESM.zip › SOURCE_DATA/Figure5_SOTA/5a_evaluation/fig5a.png]

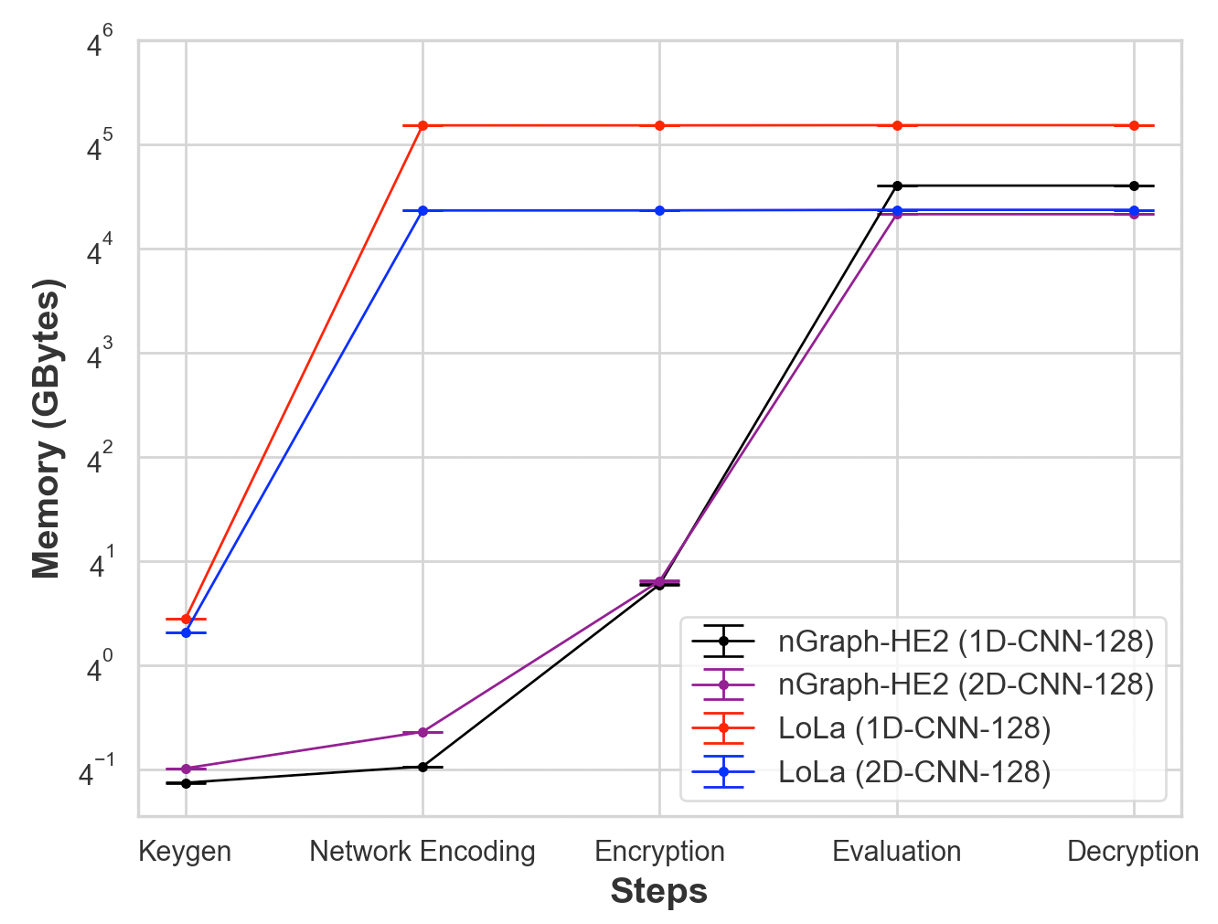

Supplement: Supplementary file 4 — Source data [file 41467_2022_32168_MOESM4_ESM.zip › SOURCE_DATA/Figure5_SOTA/5de_memory/fig5e.png]

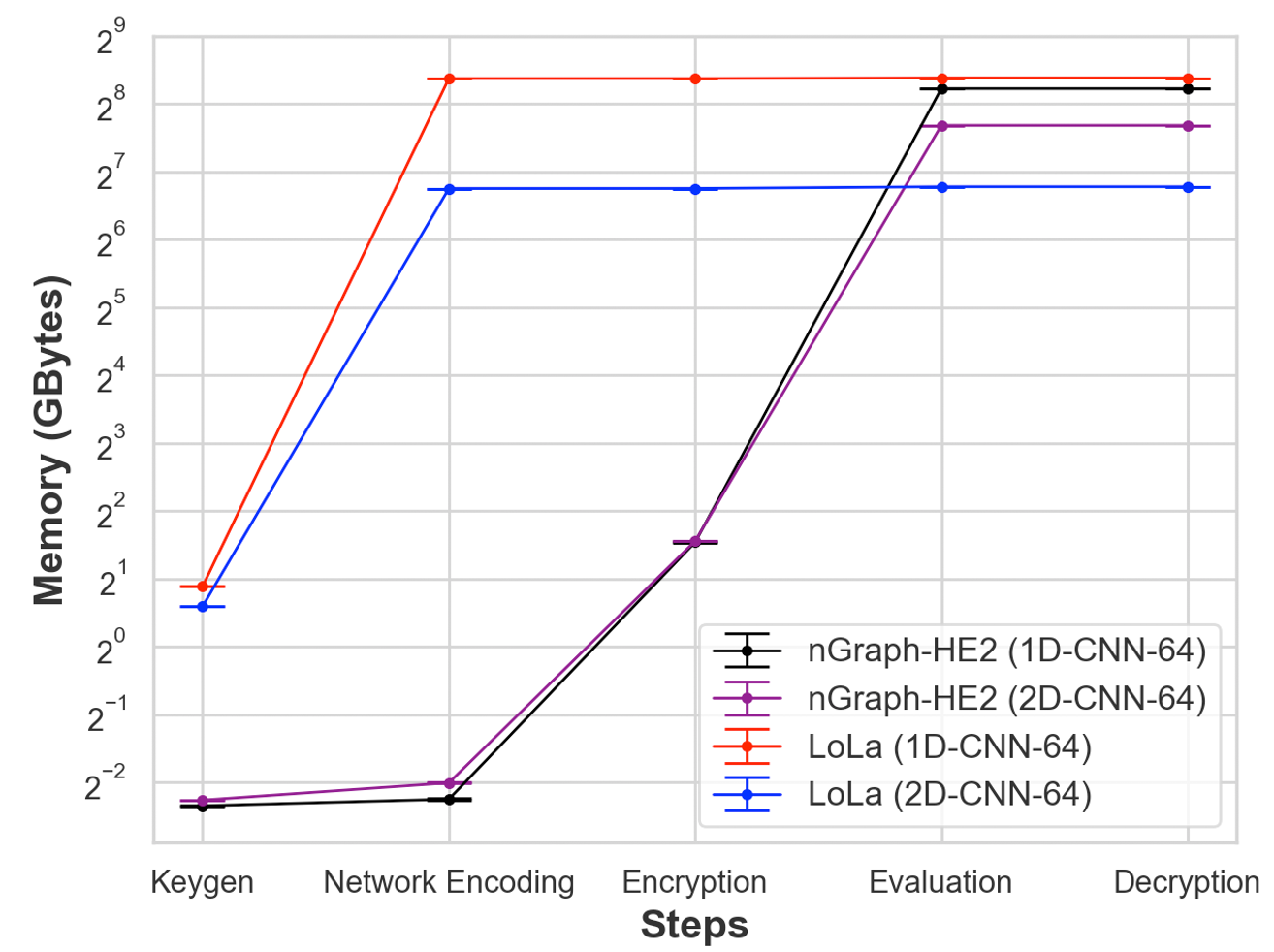

Supplement: Supplementary file 4 — Source data [file 41467_2022_32168_MOESM4_ESM.zip › SOURCE_DATA/Figure5_SOTA/5de_memory/fig5d.png]

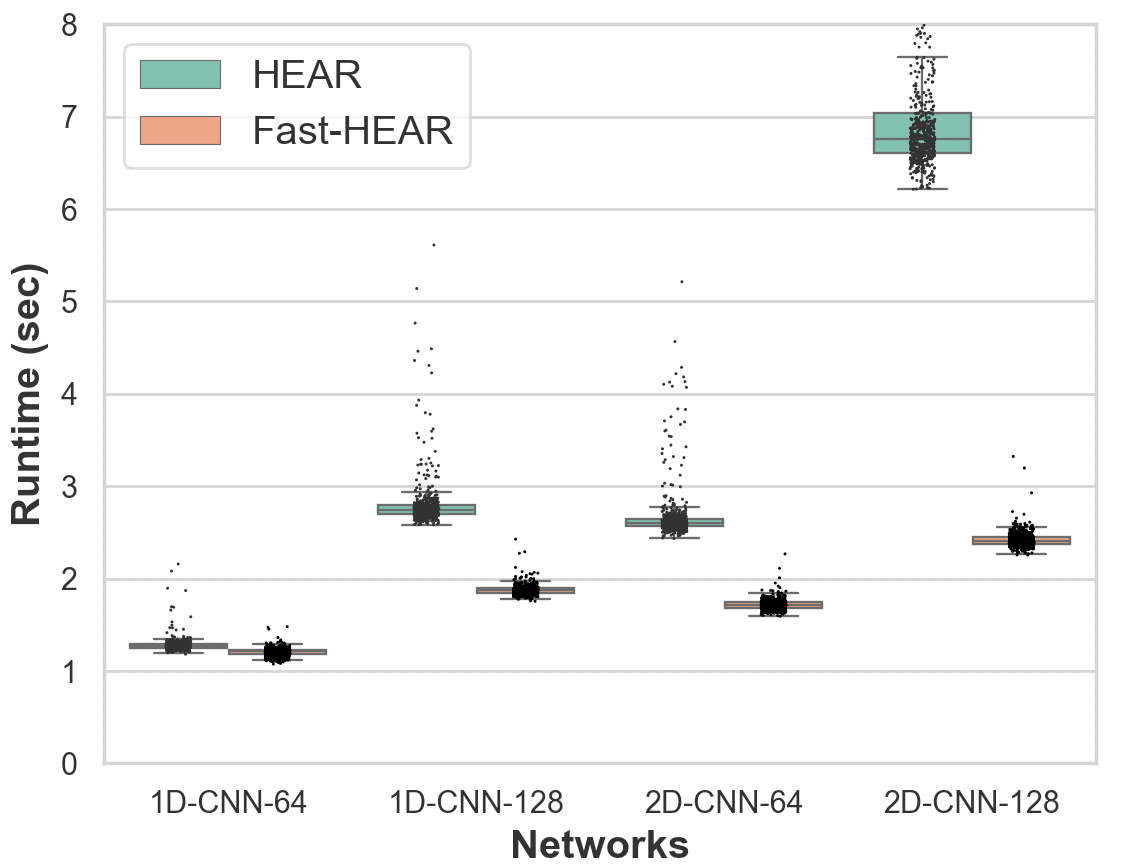

Supplement: Supplementary file 4 — Source data [file 41467_2022_32168_MOESM4_ESM.zip › SOURCE_DATA/Figure4_HEAR/4c_evaluation/fig4c.png]

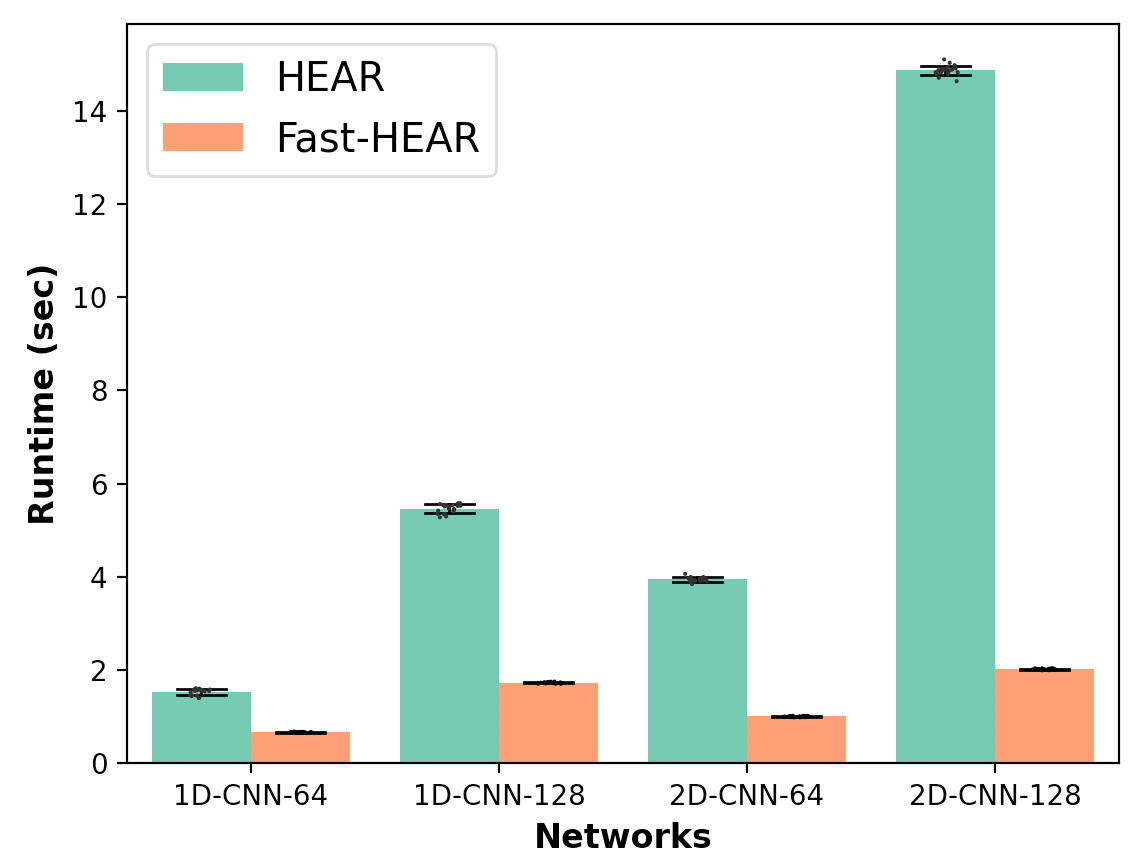

Supplement: Supplementary file 4 — Source data [file 41467_2022_32168_MOESM4_ESM.zip › SOURCE_DATA/Figure4_HEAR/4ab_keygen_encode/fig4b.png]

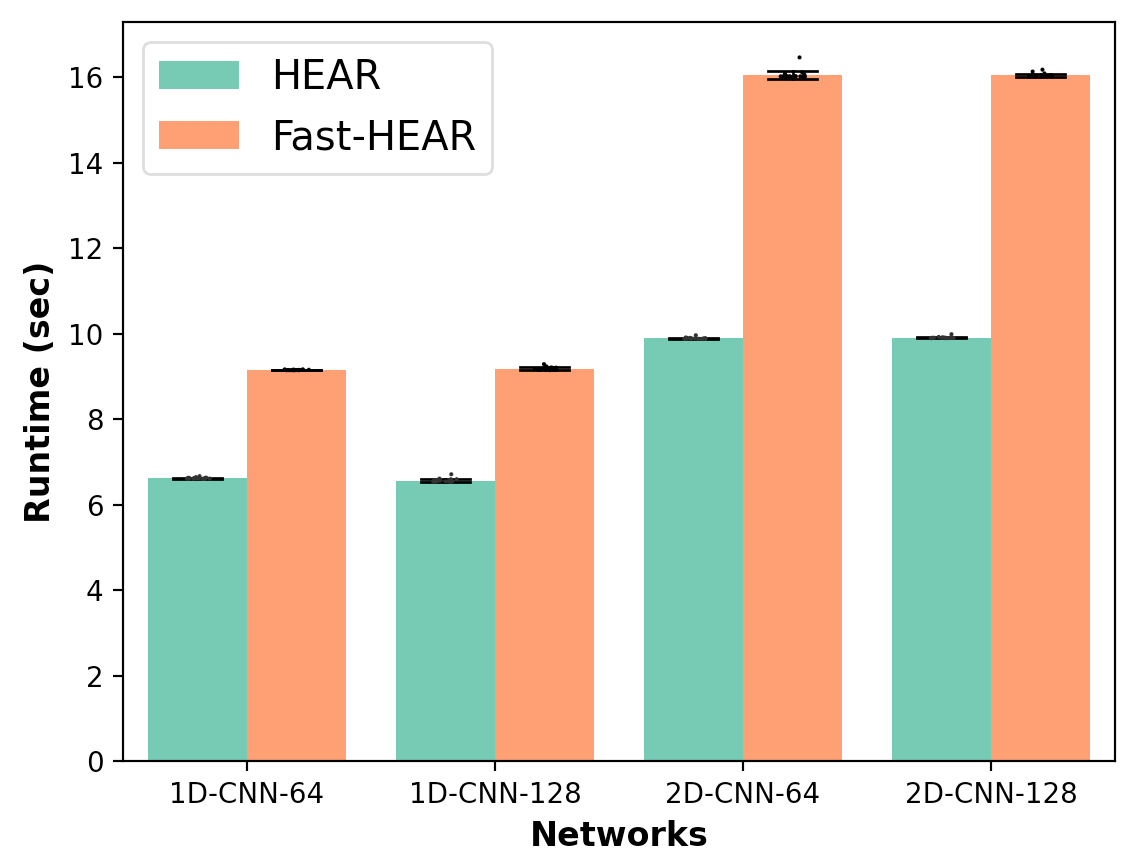

Supplement: Supplementary file 4 — Source data [file 41467_2022_32168_MOESM4_ESM.zip › SOURCE_DATA/Figure4_HEAR/4ab_keygen_encode/fig4a.png]

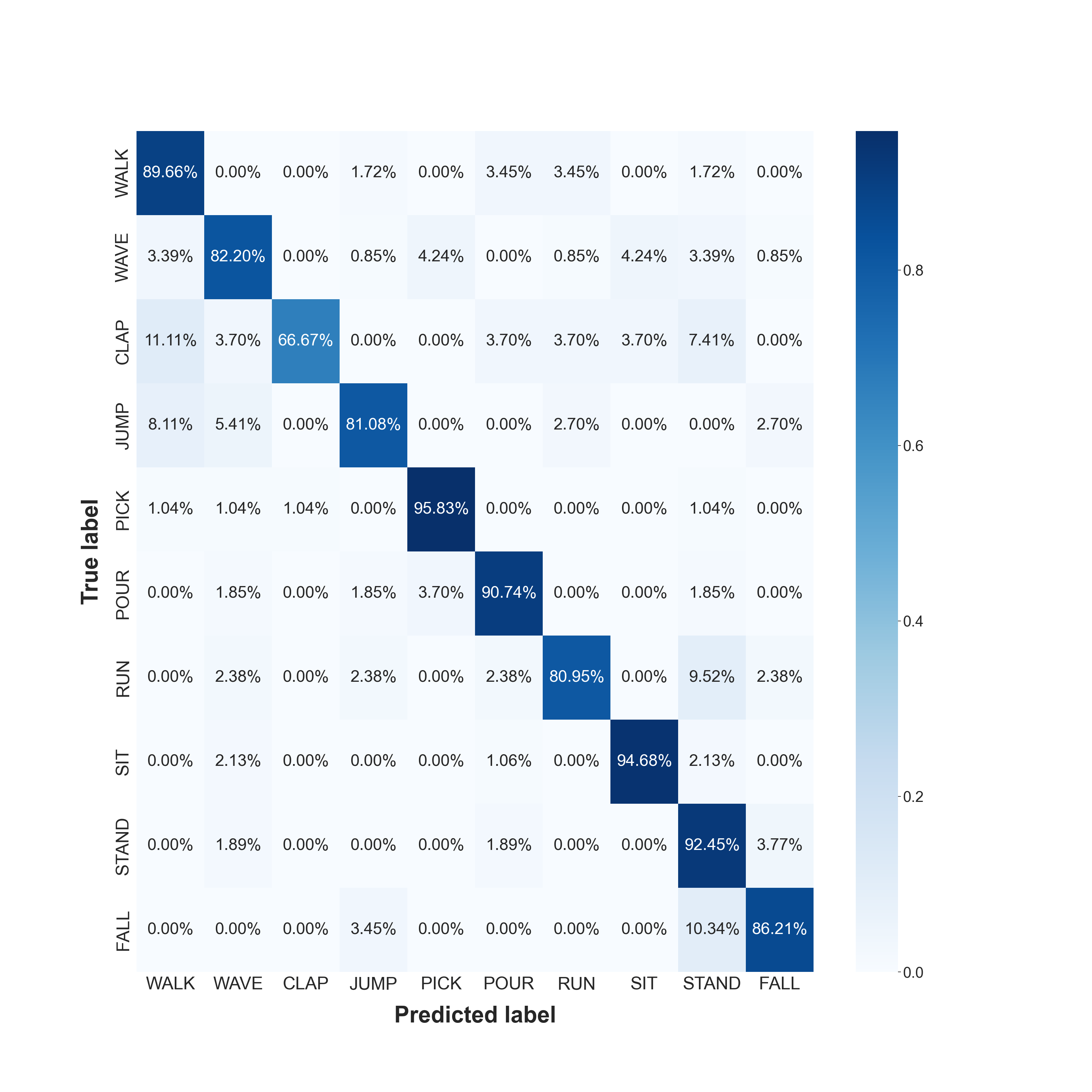

Supplement: Supplementary file 4 — Source data [file 41467_2022_32168_MOESM4_ESM.zip › SOURCE_DATA/Figure4_HEAR/4gh_confusion_matrix/confusion_matrix_fasthear.png]

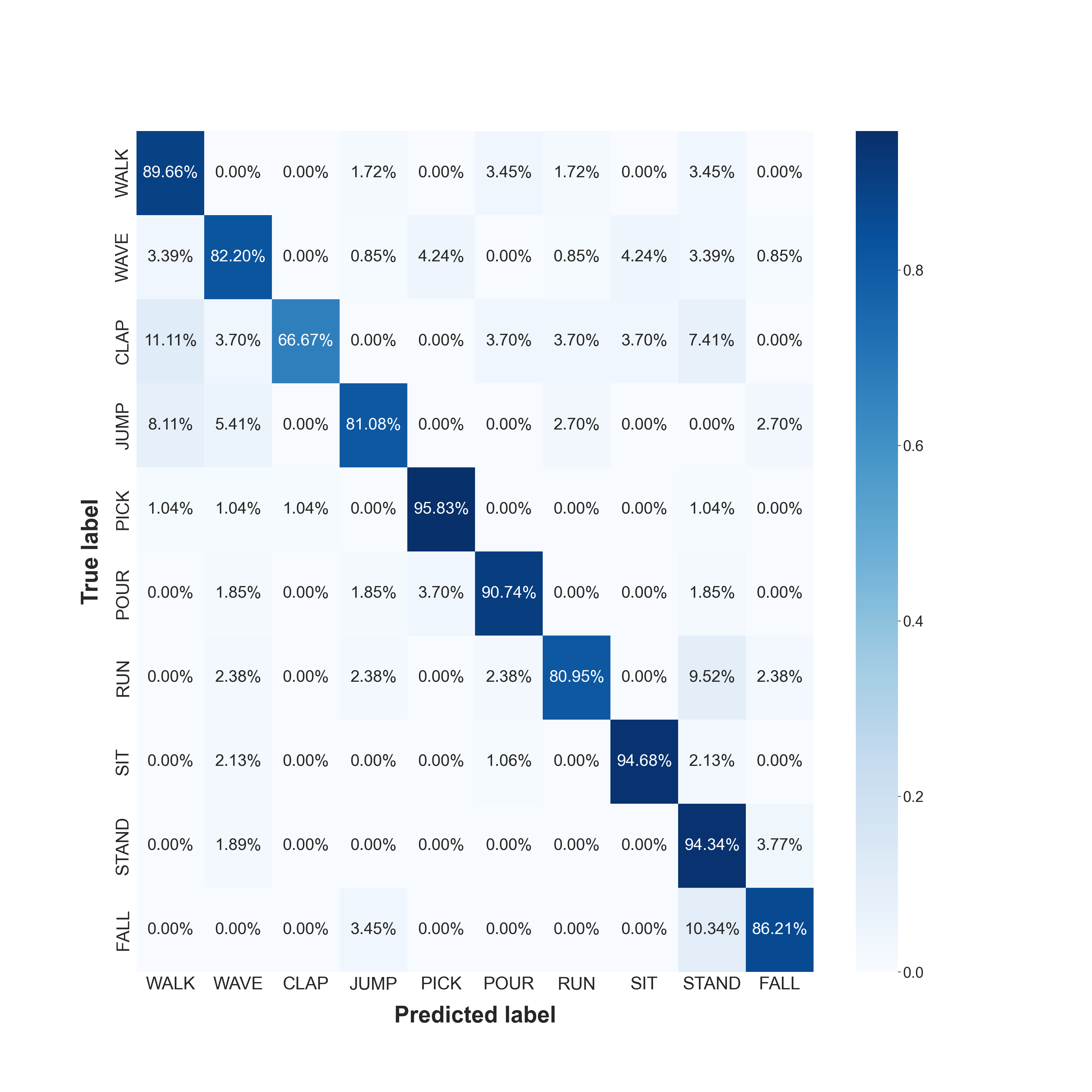

Supplement: Supplementary file 4 — Source data [file 41467_2022_32168_MOESM4_ESM.zip › SOURCE_DATA/Figure4_HEAR/4gh_confusion_matrix/confusion_matrix_plain.png]

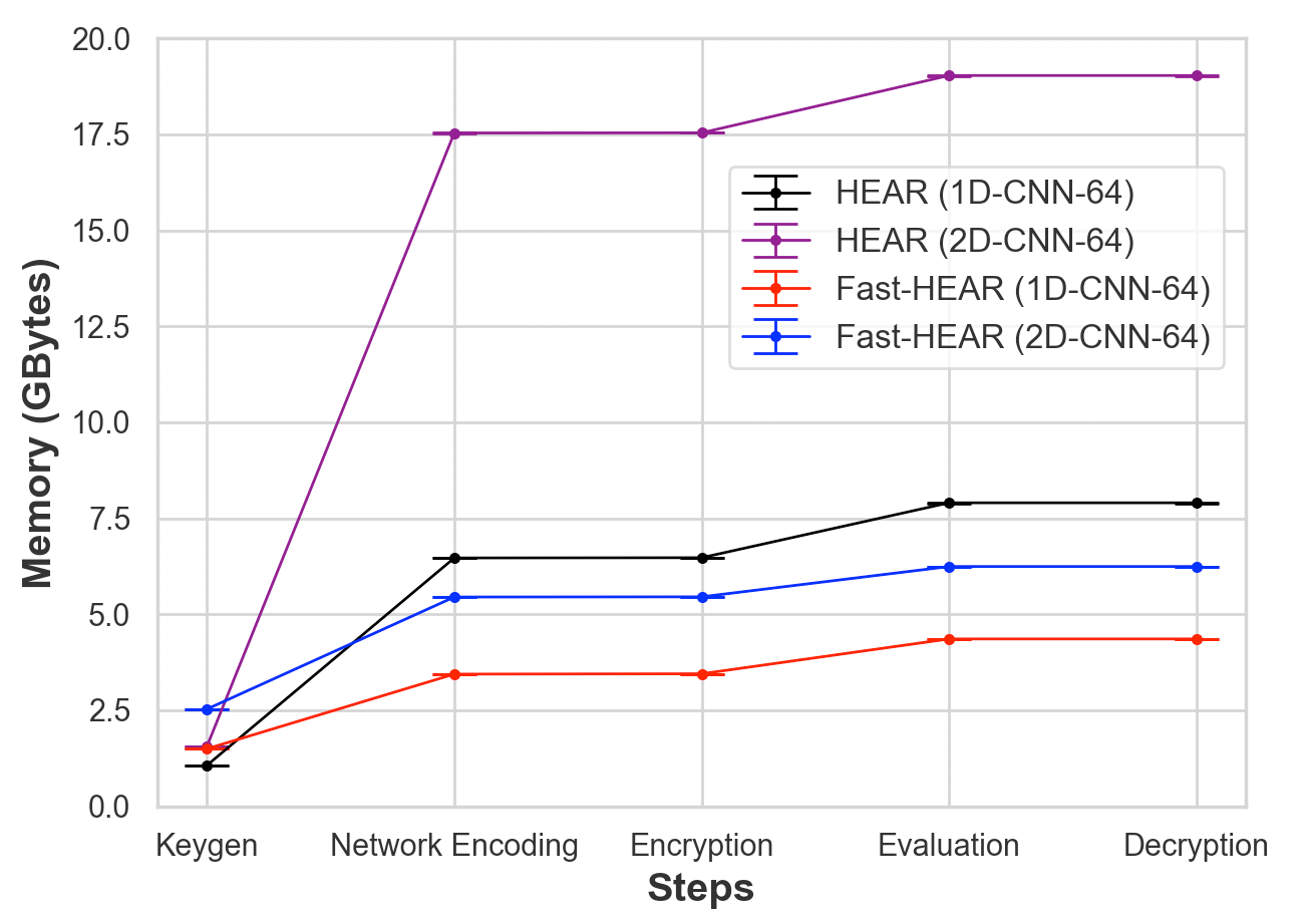

Supplement: Supplementary file 4 — Source data [file 41467_2022_32168_MOESM4_ESM.zip › SOURCE_DATA/Figure4_HEAR/4de_memory/fig4d.png]

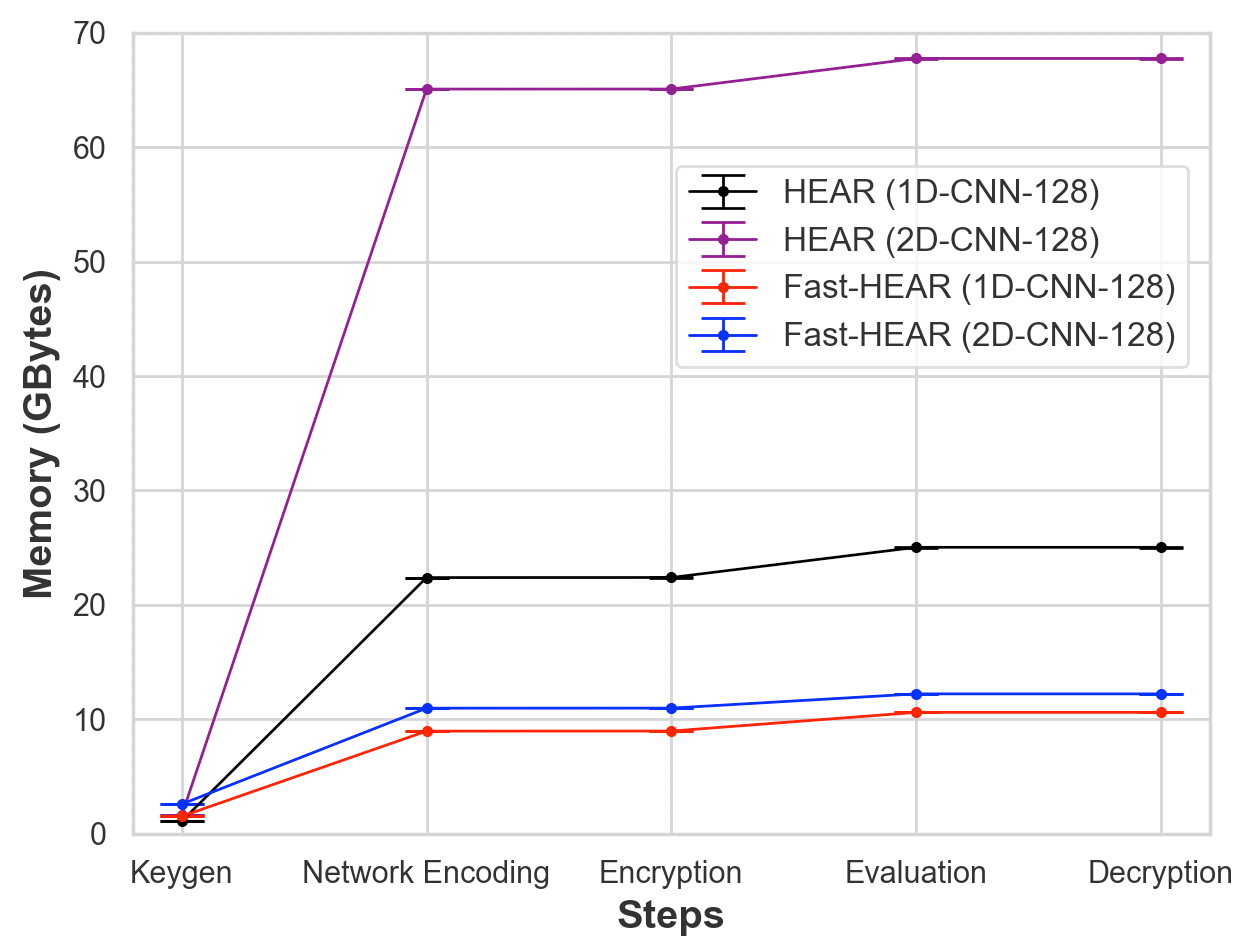

Supplement: Supplementary file 4 — Source data [file 41467_2022_32168_MOESM4_ESM.zip › SOURCE_DATA/Figure4_HEAR/4de_memory/fig4e.png]
